# *Gallibacterium anatis* Emerging in Indonesia: Isolation and Molecular Characterization From Chickens in a West Java Poultry Farm

**DOI:** 10.1155/vmi/7717406

**Published:** 2026-02-24

**Authors:** Alya Amaliah, Ni Luh Putu Ika Mayasari, Ryan Septa Kurnia, Christian Marco Hadi Nugroho, Muhammad Ade Putra, Agustin Indrawati

**Affiliations:** ^1^ Animal Biomedical Sciences, School of Veterinary Medicine and Biomedical Sciences, IPB University, Bogor, 16680, Indonesia, ipb.ac.id; ^2^ Division of Medical Microbiology, School of Veterinary Medicine and Biomedical Sciences, IPB University, Bogor, 16680, Indonesia, ipb.ac.id; ^3^ Animal Health Diagnostic Unit, PT Medika Satwa Laboratoris, Bogor, 16166, Indonesia

**Keywords:** emerging disease, *Gallibacterium anatis*, PCR, phenotypic characterization, poultry

## Abstract

*Gallibacterium anatis* (*G. anatis*) is associated with decreased egg production and respiratory disorders in chickens. The presence of *G. anatis* in Indonesia has not been reported. This study aimed to identify *G. anatis* both phenotypically and genotypically in layer, broiler, and breeder chicken from farms in West Java. A total of 23 suspected gallibacteriosis cases were collected from chickens exhibiting respiratory and reproductive disorders between May 2020 and October 2023. Phenotypic characterization was conducted using blood agar culture, Gram staining, and biochemical tests (catalase, oxidase, and TSIA). Genotypic identification of *G. anatis* was performed using PCR targeting the 16S‐23S rRNA gene, followed by detection of virulence genes (*gtxA, flfA,* and *gyrB*). Sequencing and phylogenetic analyses were carried out using BLAST program and MEGA 7. Among the 23 collected samples, 17 samples showed phenotypic characteristics of *G. anatis*, and 14 of these isolates (73.9%) were confirmed molecularly. Virulence genes profiling revealed the presence of *gyrB* (100%), *gtxA* (93.3%), and *flfA* (71.4%). Sequence analysis demonstrated more than 98.65% similarity between all isolates and reference *G. anatis* strains in GenBank, supporting accurate species identification. This study provides the molecular evidence of *G. anatis* in layer, breeder, and broiler chickens from farms in West Java, Indonesia. The detection of key virulence genes further indicates the pathogen’s potential role in reduced production and respiratory disease in Indonesian poultry. These findings highlight the importance of routine surveillance and molecular diagnostics to support early detection and control strategies for gallibacteriosis in commercial poultry populations.

## 1. Introduction

*Gallibacterium anatis* (*G. anatis*) is a bacterium that causes gallibacteriosis [1], which plays a role in reducing egg production in laying hens by up to 3%–18%. *G. anatis* is increasingly recognized as an important pathogen associated with reduced egg production and reproductive tract lesions in laying hens [2]. Infections caused by this bacterium can be primary or secondary [3]. Besides targeting the reproductive organs, this bacterium is also present in various other organs such as the heart, spleen, upper respiratory tract, lower genital tract, and multiple systemic tissues of affected chickens, reflecting its broad tissue tropism [2–5].

Historically, *G. anatis* was classified as *Pasteurella haemolytica;* however, in 2003, it was reclassified into a new genus, *Gallibacterium* [4]. In recent years, reports of *G. anatis* infections have increased worldwide, indicating its rising clinical importance [2]. A new taxonomy of *G. anatis* has been identified in several countries [6]. However, there have been no documented reports on the occurrence of *G. anatis* in Indonesia.

Detection of *G. anatis* can be performed phenotypically and genotypically. Molecular identification remains essential due to its phenotypic similarity to other respiratory pathogens [2]. Genotypic detection typically uses PCR targeting the 16S and 23S rRNA genes, and identification can be confirmed with MALDI‐TOF; indeed, isolates from reproductive, respiratory, and serosal lesions have been characterized using these methods [4]. Classification using 16S rRNA gene targets is currently widely used in bacterial diagnostics [6]. Genomic analyses have shown considerable diversity among *G. anatis* isolates, including variation in virulence‐associated genes. Some studies report highly conserved RTX toxin systems as core virulence mechanisms [2, 7], while others identified multiple antibiotic resistance genes varying between organs and flocks [8, 9]. In Morocco, *G. anatis* was isolated from ovaries, trachea, and cloaca in layer chickens with decreased egg production, indicating its direct impact on productivity [5].

Recent epidemiological studies further underscore the significance of *G. anatis*. Surveys of poultry flocks in Poland (2022–2023) found a prevalence of 22.5%, with 20% of isolates exhibiting resistance to at least eight antibiotics [10]. All *G. anatis* biovar haemolytica strains carried the toxin gene *gtxA* and showed multidrug resistance [9], while additional studies revealed widespread resistance to 20 antimicrobials across respiratory, reproductive, and gastrointestinal isolates [11]. Moreover, *G. anatis* has been detected in pet birds (∼10.5% prevalence), with isolates showing both high antibiotic resistance and moderate biofilm formation, indicating persistence and treatment challenges [12].

West Java is a province that serves as a center for chicken farming in Indonesia [13], thus serving as a starting point for identifying potentially harmful infectious agents in the poultry industry. Information regarding the presence of *G. anatis* has not yet been reported in West Java or Indonesia. Therefore, the aims of this study were to isolate and identify the bacterium *G. anatis* phenotypically and genotypically on chicken farms in West Java, to molecularly characterize the virulence genes of *G. anatis*, and to analyze the genetic relationship among *G. anatis* bacteria.

## 2. Materials and Methods

### 2.1. Ethical Approval

Ethical approval on this study was obtained from Animal Ethics School of Veterinary Medicine and Biomedical Science, IPB University with number: 121/SKE/X/2023.

### 2.2. Sample Collection

The research samples consisted of a total of 23 suspected gallibacteriosis cases in West Java from May 2020 to October 2023. The field samples were obtained from several chicken flocks across five different farms that experienced respiratory disorders, accompanied by a 7%–12% reduction in egg production and abnormalities in eggshell formation. All isolates were obtained from tracheal swab, trachea, lung, ovary, and oviduct organs of broilers, layers, and breeder chickens that were suspected of having clinical symptoms of *G. anatis* infection. Organ samples were placed in sterile plastic, while swab samples were placed in Amies transport medium (Labware), which was then transported using a cold box not exceeding 48 h from sample collection to prevent bacterial growth.

### 2.3. Isolation and Identification of *G. anatis*

Samples were obtained from tracheal swabs and reproductive and respiratory organs of chickens exhibiting respiratory disorders or decreased production. Cultures were conducted on blood agar media from archived isolates and field samples, followed by incubation at 37°C for 24–48 h. Colonies were observed macroscopically and bacterial morphology was observed microscopically by Gram staining. Biochemical tests were performed to confirm *G. anatis* by the catalase test, the oxidase test, and triple sugar iron agar (TSIA) [14].

Isolates exhibiting the phenotypic characteristics of *G. anatis* were further molecularly confirmed. Confirmation of *G. anatis* was conducted using the 16S rRNA gene target 1133fgal (5′‐TATTCTTTGTTACCARCGG‐3′) as the forward primer and 23S rRNA 114r (5′‐GGT​TTC​CCC​ATT​CGG‐3′) as the reverse primer, which yielded a positive result for *G. anatis* at a band size of 1030 bp [14].

### 2.4. Detection of Virulence Genes of *G. anatis*

All samples confirmed as *G. anatis* by PCR testing were further subjected to the detection of virulence genes of *G. anatis* bacteria encoding *gtxA* (cytolytic–hemolytic gene), *flfA* (flagellar gene), and *gyrB* (gyrase subunit B). The primer sequences used are listed in Table 1.

**TABLE 1:** Specific primers used for polymerase chain reaction (PCR) to detect virulence genes of *Gallibacterium anatis*.

Target genes	Sequences (5′–3′)	Size (bp)	References
*gtxA*	F: CAA​ACC​TAA​TTC​AAT​CGG​ATG	1257	[9]
	R: TGC​TTC​AAT​AAT​TTT​CCA​TTT​TC		

*flfA*	F: CAC​CAT​GGG​TGC​ATT​TGC​GGA​TGA​TCC	538	[9]
	R: ATT​CGT​ATG​CGA​TAG​TAT​AGT​TC		

*gyrB*	F: TGT​GCG​TTT​CTG​GCC​AAG​TC	561	[10]
	R: CGC​TCA​CCA​ACT​GCA​GAT​TC		

### 2.5. Sequence and Phylogenetic Analyses

Eight isolates of *G. anatis* were utilized for sequencing analysis using Sanger sequencing with BigDye Terminator V3.1 (Thermo Fisher Scientific GmBH, Dreieich 63303, Germany) [15]. The nucleotide sequence of the *G. anatis* 16S rRNA gene was analyzed using Basic Local Alignment Search Tool (BLAST) to determine its identity and homology to other isolates in the GenBank database of the National Center for Biotechnology Information (NCBI). Phylogenetic analysis was conducted using the Molecular Evolutionary Genetics Analysis (MEGA) 7 application with the neighbor‐joining method [16]. Phylogenetic trees were constructed by selecting reference isolates with the highest BLAST analysis scores.

## 3. Results

### 3.1. Phenotypic and Genotypic Identification of *G anatis*

A total of 17 (73.9%) of 23 samples showed suspected growth of *G. anatis.* All of these demonstrated growth as small‐sized, nonmucoid, semitransparent colonies with a distinct hemolytic zone on blood agar (Figure 1). Morphological test results are Gram‐negative and pleomorphic coccobacilli. Biochemical test results were catalase‐positive, oxidase‐positive, and indole‐negative.

FIGURE 1Cultural and microscopic characteristics of *Gallibacterium anatis*. (a) Growth of *G. anatis* on blood agar. (b) Gram‐stained *G. anatis* demonstrating Gram‐negative coccobacilli under 1000× magnification.

**Figure:**
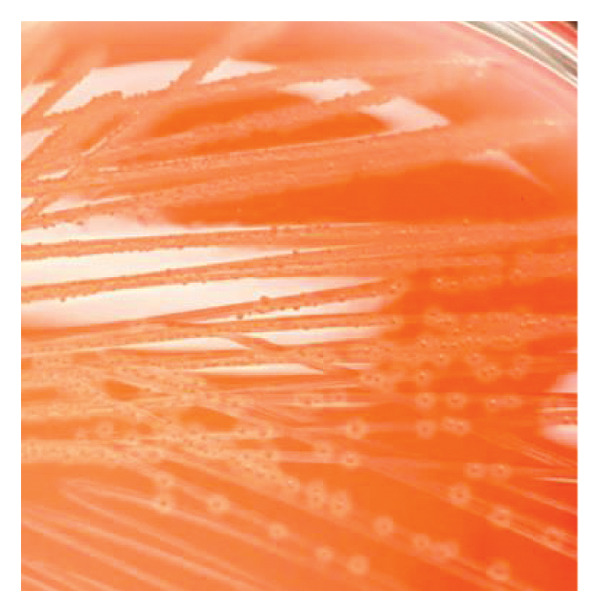
(a)

**Figure:**
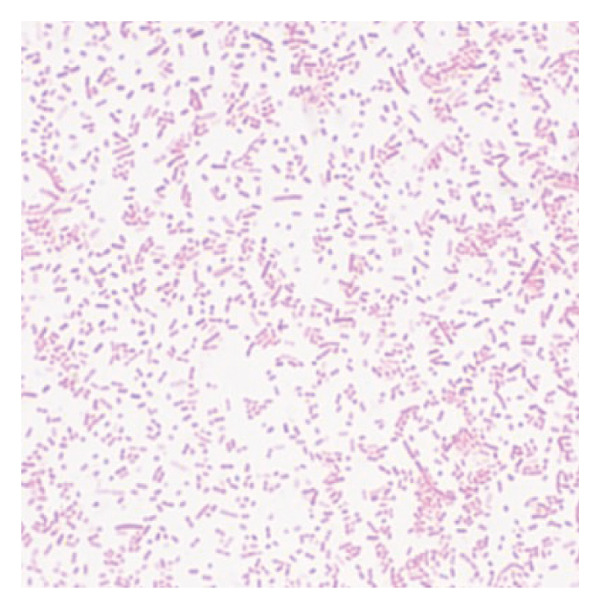
(b)

Identification of 17 positive *G. anatis* samples based on phenotypic observation consisted of five sample isolates from a breeder–layer farm, four isolates from broiler farms in Cibinong, and eight isolates from layer farms in Cibinong (Table 2). Molecular confirmation of suspected *G. anatis* isolates showed that, out of the three layer chicken farms, two farms were confirmed to have *G. anatis* (Table 2). The farms confirmed to have *G. anatis* experienced a decrease in egg production ranging from 5% to 20%. *G. anatis* bacteria were identified in chicken farms in the same area, specifically in the Cibinong region. On the broiler chicken farm, out of the two farms sampled, one farm was confirmed to have *G. anatis*, with a positivity rate of 75% in chickens experiencing respiratory disturbances ranging from moderate to severe.

**TABLE 2:** Number of *Gallibacterium anatis* isolates from West Java during the period 2020–2023.

Origin of isolates	Number of samples	Number of phenotypic test	Number of molecular test	
			Positive	Negative
Farm A (Breeder‐Layer)	5	5 (100%)	5 (100%)	0 (0%)
Farm 1 (Layer‐Ciampea)	3	0 (0%)	—	—
Farm 2 (Broiler‐Ciseeng)	3	0 (0%)	—	—
Farm 3 (Broiler‐Cibinong)	4	4 (100%)	3 (75%)	1 (25%)
Farm 4 (Layer‐Cibinong)	4	4 (100%)	3 (75%)	1 (25%)
Farm 5 (Layer‐Cibinong)	4	4 (100%)	3 (75%)	1 (25%)
Total	23	17 (73.9%)	14 (82.35%)	6 (17.64%)

### 3.2. Presence Virulence Genes of *G. anatis*

**FIGURE 2:**
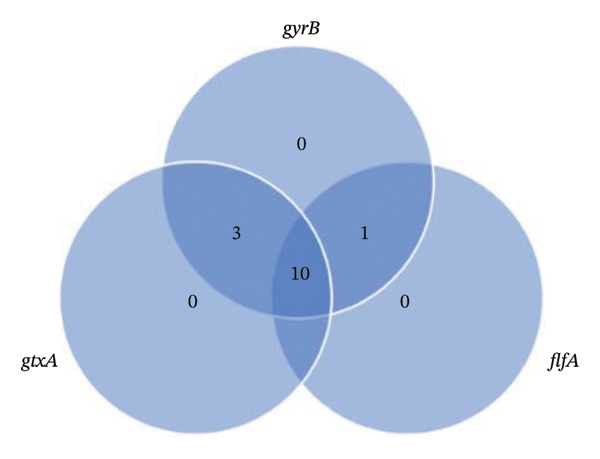
Distribution patterns of *Gallibacterium anatis* virulence genes. Each circle is representatives of the virulence gene, such as g*txA, flfA, and gyrB*.

Virulence gene analysis was conducted targeting three genes: *gtxA, gyrB*, and *flfA*. Among the 14 confirmed *G. anatis* isolates, a positive result for *gtxA* was detected in 93.3% (13/14), for *gyrB* in 100% (14/14), and for *flfA* in 71.4% (10/14) of the isolates (Table 3). Three isolates were negative for the *flfA* gene, originating from Sukabumi (broiler, breeder, and layer chicken) and Bogor (layer chicken). Only one isolate (002/BELY/CS–BG/2022) tested negative for *gtxA*. Analysis of the *G. anatis* virulence gene combinations showed that each isolate carried at least two virulence genes (28.57%), while the highest prevalence (71.42%) was observed in isolates harboring all three virulence genes (Figure 2).

**TABLE 3:** Number of virulence genes tested in *Gallibacterium anatis*.

Isolates name	Virulence genes		
	*gtxA*	*gyrB*	*flfA*
001/BEBR/SBM/2020	+	+	−
002/BELY/CS–BG/2022	−	+	+
003/BELY/CS–BG/2022	+	+	+
004/BEBR/PR–BG/2022	+	+	+
005/LY/SBM/2022	+	+	−
012/BR/CB–BG/2023	+	+	+
014/BR/CB–BG/2023	+	+	+
015/BR/CB–BG/2023	+	+	+
016/LY/CB–BG/2023	+	+	+
017/LY/CB–BG/2023	+	+	+
018/LY/CB–BG/2023	+	+	+
020/LY/CB–BG/2023	+	+	+
021/LY/CB–BG/2023	+	+	−
022LY/CB–BG/2023	+	+	+

*Note:* BEBR = breeder broiler; BELY = breeder layer; LY = layer; BR = broiler; CS = Cisarua; BG = Bogor; PR = Parung; SBM = Sukabumi; CB = Cibinong.

### 3.3. Similarity and Phylogenetic Tree of *G. anatis*

Similarity analysis using BLAST revealed that the 16S rRNA gene sequence of the isolates were highly similar to *G. anatis*. Similarity values greater than 98.65% indicate that a bacterial isolate belongs to the same species, while values above 95% can identify the genus [17]. In this study, all nucleotide sequences showed a similarity exceeding 98.65% with *G. anatis* (Table 4).

**TABLE 4:** Results of similarity of *Gallibacterium anatis* 16S‐23S rRNA.

Isolates name	Bacterial references	Access code	Similarity	Query cover
001/BEBR/SBM/2020	*G. anatis* strain ESV200	CP114281.1	99.38%	100%

002/BELY/CS–BG/2022	*G. anatis* strain ESV 200	CP114281.1	99.64%	100%
	*G. anatis* strain YU‐ZMD	GU475462.1	99.64%	100%

003/BELY/CS–BG/2022	*G. anatis* strain TH22	CP103874.1	99.53%	100%
	*G. anatis* strain YJ922	CP126978.1	99.53%	100%
	*G. anatis* strain YU‐ZMD	GU475462.1	99.53%	100%

004/BEBR/PR–BG/2022	*G. anatis* strain ESV200	CP114281.1	99.27%	100%

005/LY/SBM//2022	*G. anatis* strain TH22	CP103874.1	99.73%	100%
	*G. anatis* strain DFSO2	CP126977.1	99.73%	100%
	*G. anatis* strain YJ922	CP126978.1	99.73%	100%
	*G. anatis* strain BJF12	CP126975.1	99.73%	100%
	*G. anatis* strain ESV200	CP114281.11	99.73%	100%
	*G. anatis* strain UMN179	CP002667.1	99.73%	100%

013/BR/CB–BG/2023	*G. anatis* strain TH22	CP103874.1	99.62%	100%
	*G. anatis* strain DFSO2	CP126977.1	99.62%	100%
	*G. anatis* strain YJ922	CP126978.1	99.62%	100%
	*G. anatis* strain YU‐ZMD	GU475462.1	99.62%	100%

016/LY/CB–BG/2023	*G. anatis* strain TH22	CP103874.1	99.52%	100%
	*G. anatis* strain BJF12	CP126975.1	99.52%	100%
	*G. anatis* strain YU‐ZMD	GU475462.1	99.52%	100%

017/LY/CB–BG/2023	*G. anatis* strain IMT49310	CP110225.1	99.75%	100%

*Note:* BEBR = breeder broiler; BELY = breeder layer; LY = layer; BR = broiler; CS = Cisarua; BG = Bogor; PR = Parung; SBM = Sukabumi; CB = Cibinong.

The bacterial isolates analyzed here shared similarities with strains from various countries. Specifically, strains YU‐ZMD, TH22, YJ922, DFS02, and BJF12 originated from chickens in China; ESV200 from Mexico; UMN179 from the United States; and IMT49310 from Germany.

Confirmation of the taxonomic proximity of a bacterial isolate by using similarity values can be reinforced by constructing a phylogenetic tree. The phylogenetic tree showed that all isolates clustered within the *G. anatis* in‐group, forming two main supergroups, while three reference isolates from China and the United States formed a different supergroup. Outgroup species, *G. salpingitidis* and *G. trehalosifermentans* formed distinct clusters but remained within the overall *Gallibacterium* clade.

**FIGURE 3:**
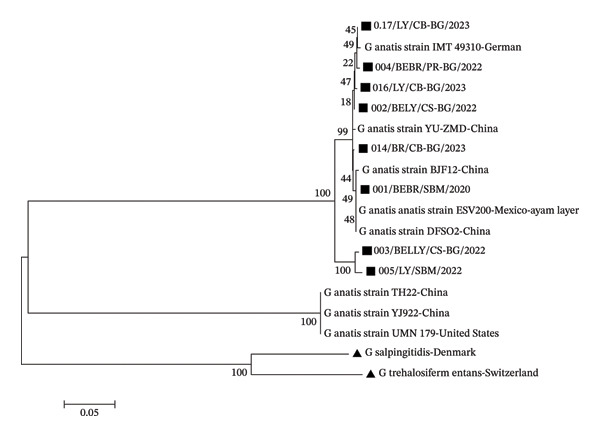
Phylogenetic tree of *G. anatis* isolates from Indonesia. This phylogenetic tree was constructed using neighbor‐joining with the MEGA 7 application from the 16S‐23S rRNA gene. The branches of the dendrogram show bootstrap values (%) with 1000 replications, and the scale represents one per 1000 nucleotide sequence substitutions of the 16S rRNA gene. () denotes the isolates tested. () denotes outgroup reference isolates.

The phylogenetic tree also indicated that isolates 003/Breeder Layer/Cisarua–Bogor/2022 and 005/Layer/Sukabumi//2022 were more closely related, supported by a bootstrap value of 100% (Figure 3). However, other isolates showed variable relationships with both the tested and reference strains, with bootstrap values below 70%. Bootstrap values reflect the reliability of the phylogenetic tree, where values greater than 70% correspond to a confidence level exceeding 95% [18]. The highest bootstrap values in some relationship suggest that the formed tree could vary if alternative phylogenetic reconstruction methods are applied.

## 4. Discussion

In the present study, *G. anatis* was successfully isolated from poultry farms in West Java, Indonesia. Notably, the presence of *G. anatis* has also been identified in Europe, Africa, China, India, Japan, North America, and South America [6], underscoring its global distribution. Recent reports further confirm its widespread emergence in commercial poultry systems, particularly in Asia and Europe, where multidrug‐resistant and highly virulent strains have been documented [9]. *G. anatis* can cause a decrease in egg production in layer and breeder chickens, as well as respiratory disorders in broilers. In this study, *G. anatis* was identified in layer farms in the Cibinong area. This is consistent with the findings of Yaman and Yapicier [6], who reported *G. anatis* in Turkish layer farms experiencing decreased egg production. A decrease in production was also observed on Farm 1 in the Ciampea region, but *G. anatis* was not identified. Such production drops may be linked to other bacterial or viral pathogens that were not identified in this study. Multifactorial drops in egg production have been frequently reported in the field, as coinfections with *E. coli*, *Mycoplasma* spp., *Avibacterium paragallinarum*, or viral pathogens often complicate accurate attribution of production losses [19, 20].

The molecular results for *G. anatis* in this study indicated that not all isolates showing phenotypic suspicion of *G. anatis* were successfully molecularly confirmed using the 16S‐23S rRNA gene. One sample from Farms 3, 4, and 5 showed negative results based on molecular analysis. This could be due to the fact that phenotypic testing relies on biochemical pathways and carbon source utilization, which may have similarities with other bacteria, especially within the same family. Bacteria with phenotypic characteristics similar to those of *G. anatis* include *Pseudomonas aeruginosa* and *Mannheimia haemolytica* [21]. These results highlight the importance of combining phenotypic and molecular methods for accurate identification. This challenge aligns with recent findings showing that *G. anatis* displays substantial phenotypic variability, which can lead to misidentification when relying solely on biochemical assays [22].

The virulence genes of *G. anatis* have previously been identified. The *flfA* gene encodes the fimbriae in *G. anatis* and plays a role in the adhesion process, especially in the oropharyngeal epithelial cells of chickens, which have three fimbrial gene clusters [23, 24]. The presence of *flfA* can be associated with tissue tropism [25], but the data in this study are not sufficient to establish this correlation. The upper respiratory tract, particularly the trachea, is the common site of *G. anatis* colonization [2]. However, *G. anatis* has been detected in internal organs such as the liver, heart, and lungs [14, 15]. These findings suggest that certain strains have the potential for extra‐respiratory dissemination, although the mechanisms underlying this spread remain to be fully elucidated.

The *gtxA* gene encodes a toxin that can cause inflammation and is responsible for hemolytic activity [26]. *G. anatis* bacteria are grouped into two biotypes: hemolytic and nonhemolytic. The presence of the *gtxA* gene can be used to classify isolates as belonging to the hemolytic biotype. This classification can also be observed through phenotypic testing, which results in hemolytic zones on blood agar media. In this study, Isolate 002/BELY/CS‐BG/2022 exhibited a hemolytic zone but tested negative for the *gtxA* gene. This is likely due to the low concentration of the *gtxA* gene in the medium during the late log phase of the bacterial growth cycle [27], making it undetectable in molecular testing. Additionally, other isolates that displayed hemolytic zones on blood agar correlated with the presence of the *gtxA* gene in this test. Similar discrepancies have been reported in recent studies, where *gtxA* expression was shown to be variable based on the growth phase and environmental conditions, leading to inconsistent molecular detection despite clear phenotypic hemolysis [11].

All isolated samples tested positive for encoding the *gyrB*, which is associated with virulence. The presence of *gyrB* in these isolates is significant because the gene encodes the ATPase domain of gyrase, which is essential for bacterial DNA replication [28]. The *gyr*B gene can be used as a discriminatory marker for identifying *G. anatis* and differentiating closely related *Pasteurellaceae* species [22, 29]. The presence of these virulence genes confirms the pathogenic potential of the isolates collected in this study.

Similarity analysis showed that Isolates 016/LY/CB–BG/2023 and 017/LY/CB–BG/2023 originated from the same type of chicken and area; however, they exhibited different reference bacterial strains based on similarity values. Isolate 014/BR/CB–BG/2023, the only broiler chicken tested in this study, showed similar testing results that can also be found in isolates originating from layer chickens or breeders. Interestingly, Isolate 017/LY/CB‐BG/2023, from layer chickens, showed high similarity to isolates from different host species, including cattle. This suggests that the 16S–23S rRNA gene is highly conserved and may not reflect host‐specific adaptation, indicating that sequence similarity does not necessarily correspond to the original host of the isolate. This is consistent with recent genomic studies showing that *G. anatis* forms host‐independent lineages and that cross‐species similarities are common, especially between poultry and ruminant isolates [9].

Phylogenetic analysis of *G. anatis* has been conducted extensively to classify this bacterium. Partial use of the 16S‐23S rRNA gene was unable to differentiate isolates based on their origin or epidemiology. Phylogenetic analysis using the 16S‐23S rRNA gene can only group isolates within the same species but not necessarily within the same group with good bootstrap values. In addition to using this gene, the *flfA* gene has also been utilized for bacterial analysis; however, it also fails to differentiate this gene based on sample origin. Current evidence strongly supports the use of multilocus sequence typing (MLST), core‐genome SNP analysis, or whole‐genome sequencing (WGS) to obtain clearer cluster separation and better infer transmission routes and virulence‐associated lineages [7].

These findings demonstrate that *G. anatis* is present in West Java poultry farms, with isolates carrying virulence genes that may contribute to decreased egg production and respiratory disorders. Future studies should include larger sample sizes, additional virulence or housekeeping genes, and WGS to better understand the epidemiology, host adaptation mechanisms, and pathogenicity of *G. anatis* in Indonesian poultry systems.

## 5. Conclusion

This is the first report to publish findings regarding the presence of *G. anatis* isolates in Indonesia. A total of 17 isolates were phenotypically identified as *G. anatis.* Molecular analysis confirmed that 14 of these isolates were *G. anatis*, and the virulence genes *gtxA*, *gyrB*, and *flfA* were detected, showing diverse patterns. Nucleotide analysis results indicated that the isolates had similarities to *G. anatis* with various strains. However, the *G. anatis* strain in this study could not be classified into a specific group in the phylogenetic tree constructed using the 16S‐23S rRNA gene sequence. The presence of *G. anatis* in chickens experiencing respiratory disorders or decreased production is similar to that of *G. anatis* in several countries, confirming the existence of this bacterium.

## Funding

The publication of this journal was granted by the United States Agency for International Development (USAID) throughout the SEAOHUN One Health Scholarship Program. The contents are the responsibility of the authors and do not necessarily reflect the views of USAID or the United States Government.

## Conflicts of Interest

The authors declare no conflicts of interest.

## Data Availability

The data that support the findings of this study are available from the corresponding author upon reasonable request.

## References

[1] Singh B. R. , Singh S. V. , Palanivelu M. , Kumar M. A. , Sinha D. K. , and Kumar O. R. V. , *Gallibacterium anatis* Outbreaks in Domestic Birds in North India: Antimicrobial and Herbal Drug Sensitivity of *Avibacterium* and *Gallibacterium* Isolates, IJPS. (2018) 53, no. 2, 10.5958/0974-8180.2018.00043.0.

[2] Elbestawy A. R. , Ellakany H. F. , Abd El-Hamid H. S. et al., Isolation, Characterization, and Antibiotic Sensitivity Assessment of *Gallibacterium anatis* Biovar *Haemolytica*, From Diseased Egyptian Chicken Flocks During the Years 2013 and 2015, Poul Sci. (2018) 97, 1519–1525, 10.3382/ps/pey007, 2-s2.0-85047221591. 29471426

[3] Neubauer C. , De Souza-Pilz M. , Bojesen A. M. , Bisgaard M. , and Hess M. , Tissue Distribution of Haemolytic *Gallibacterium anatis* Isolates in Laying Birds with Reproductive Disorders, Avian Pathology. (2009) 38, 1–7, 10.1080/03079450802577848, 2-s2.0-60549088915. 19089694

[4] Bojesen A. M. , Christensen H. , Nielsen O. L. , Olsen J. E. , and Bisgaard M. , Detection of *Gallibacterium* spp. in Chickens by Fluorescent 16S Rrna in Situ Hybridization, Journal of Clinical Microbiology. (2003) 41, no. 11, 5167–5172, 10.1128/JCM.41.11.5167-5172.2003, 2-s2.0-0242425745. 14605154 PMC262499

[5] Nassik S. , Tallouzt S. , Karbach N. et al., First Report of Isolation of *Gallibacterium anatis* From Layer Chickens in Morocco with Decrease in Laying Performance, Avian Diseases. (2019) 63, no. 4, 10.1637/aviandiseases-D-19-00119. 31865689

[6] Yaman S. and Sahan Yapicier O. , Diagnosis of *Gallibacterium anatis* in Layers: First Report in Turkey, Brazilian Journal of Poultry Science. (2019) 21, no. 3, 10.1590/1806-9061-2019-1019.

[7] Guo F. , Wang D. , Wu H. et al., Genomic Characteristics, Antimicrobial Resistance Profiles and Virulence Factors of *Gallibacterium anatis* Isolates from Layer Chickens in Northern China, BMC Microbiology. (2025) 25, no. 1, 10.1186/s12866-025-04440-3. PMC12581362 41184760

[8] Palmieri N. , Hess C. , and Hess M. , GWAS and Comparative Genomics Reveal Candidate Antibiotic Resistance Genes in the Avian Pathogen *Gallibacterium anatis* for Six Widespread Antibiotics, Veterinary Microbiology. (2024) 290, 10.1016/j.vetmic.2024.109995. 38301451

[9] Karwańska M. , Wieliczko A. , Bojesen A. M. , Villumsen K. R. , Krzyżewska-Dudek E. , and Woźniak-Biel A. , Isolation and Characterization of Multidrug Resistant *Gallibacterium anatis* Biovar *Haemolytica* Strains From Polish Geese and Hens, Veterinary Research. (2023) 54, no. 1, 10.1186/s13567-023-01198-2. PMC10463661 37612766

[10] Kursa O. , Tomczyk G. , Sieczkowska A. , and Sawicka-Durkalec A. , Prevalence, Identification and Antibiotic Resistance of *Gallibacterium anatis* Isolates From Chickens in Poland, Pathogens. (2023) 12, no. 8, 10.3390/pathogens12080992. PMC10458089 37623952

[11] Kursa O. , Tomczyk G. , Sieczkowska A. , and Sawicka-Durkalec A. , Antibiotic Resistance of *Gallibacterium anatis* Biovar *Haemolytica* Isolates From Chickens, Journal of Veterinary Research. (2024) 68, no. 1, 93–100, 10.2478/jvetres-2024-0007. 38525234 PMC10960332

[12] Nofouzi K. , Shakeri S. , Nikkhah S. et al., *Gallibacterium anatis* as an Emerging Pathogen in Pet Birds: Biofilm Formation Contributes to Treatment Challenges and Persistence, BMC Microbiology. (2025) 25, no. 1, 10.1186/s12866-025-04263-2. PMC12360027 40820131

[13] Syahlani S. P. , Haryadi F. T. , Setyawan A. A. , Mayasari I. , Dewi N. M. A. K. , and Qui N. H. , Key Driver of Repurchase Intention in the Poultry Farming Input Market in Indonesia, Tropical Animal Science Journal. (2022) 45, no. 4, 490–498, 10.5398/tasj.2022.45.4.490.

[14] Sorour H. K. , Atfeehy N. M. A. , and Shalaby A. G. , *Gallibacterium anatis* Infection in Chickens and Ducks, Assiut Veterinary Medical Journal. (2015) 61, 80–86, 10.21608/avmj.2015.170239.

[15] Algammal A. M. , Abo Hashem M. E. , Alfifi K. J. et al., Sequence Analysis, Antibiogram Profile, Virulence and Antibiotic Resistance Genes of XDR and MDR *Gallibacterium anatis* Isolated from Layer Chickens in Egypt, IDR. (2022) 15, 4321–4334, 10.2147/IDR.S377797. PMC9375569 35971557

[16] Kumar S. , Stecher G. , and Tamura K. , MEGA7: Molecular Evolutionary Genetics Analysis Version 7.0 for Bigger Datasets, Molecular Biology and Evolution. (2016) 33, no. 7, 1870–1874, 10.1093/molbev/msw054, 2-s2.0-85022158148. 27004904 PMC8210823

[17] Rossi-Tamisier M. , Benamar S. , Raoult D. , and Fournier P.-E. , Cautionary Tale of Using 16S Rrna Gene Sequence Similarity Values in Identification of Human-Associated Bacterial Species, International Journal of Systematic and Evolutionary Microbiology. (2015) 65, no. Pt_6, 1929–1934, 10.1099/ijs.0.000161, 2-s2.0-84938068146. 25736410

[18] Hillis D. M. and Bull J. J. , An Empirical Test of Bootstrapping as a Method for Assessing Confidence in Phylogenetic Analysis, Systematic Biology. (1993) 42, no. 2, 182–192, 10.1093/sysbio/42.2.182, 2-s2.0-12044253190.

[19] Roberts J. R. , Souillard R. , and Bertin J. , Avian Diseases Which Affect Egg Production and Quality, Improving the Safety and Quality of Eggs and Egg Products. (2011) Elsevier, 376–393, 10.1533/9780857093912.3.376, 2-s2.0-84879969509.

[20] Yehia N. , Salem H. M. , Mahmmod Y. et al., Common Viral and Bacterial Avian Respiratory Infections: An Updated Review, Poultry Science. (2023) 102, no. 5, 10.1016/j.psj.2023.102553. PMC10064437 36965253

[21] Dousse F. , Thomann A. , Brodard I. et al., Routine Phenotypic Identification of Bacterial Species of the Family *Pasteurellaceae* Isolated from Animals, Journal of Veterinary Diagnostic Investigation. (2008) 20, no. 6, 716–724, 10.1177/104063870802000602, 2-s2.0-57749168648. 18987220

[22] Huangfu H. , Xu W. , Wang H. et al., Detection of *Gallibacterium anatis* by Taqman Fluorescent Quantitative PCR, Avian Pathology. (2018) 47, no. 3, 245–252, 10.1080/03079457.2017.1416590, 2-s2.0-85041125975. 29243936

[23] Bager R. J. , Nesta B. , Pors S. E. et al., The Fimbrial Protein FlfA From *Gallibacterium anatis* is a Virulence Factor and Vaccine Candidate, Infection and Immunity. (2013) 81, no. 6, 1964–1973, 10.1128/IAI.00059-13, 2-s2.0-84877821059. 23509151 PMC3676021

[24] Kudirkienė E. , Bager R. J. , Johnson T. J. , and Bojesen A. M. , Chaperone-Usher Fimbriae in a Diverse Selection of *Gallibacterium* Genomes, BMC Genomics. (2014) 15, no. 1, 10.1186/1471-2164-15-1093, 2-s2.0-84924293478. PMC4299563 25495603

[25] Persson G. and Bojesen A. M. , Bacterial Determinants of Importance in the Virulence of *Gallibacterium anatis* in Poultry, Veterinary Research. (2015) 46, no. 1, 10.1186/s13567-015-0206-z, 2-s2.0-84931043965. PMC4462078 26063044

[26] Tang B. , Pors S. E. , Kristensen B. M. , Skjerning R. B. J. , Olsen R. H. , and Bojesen A. M. , GtxA is a Virulence Factor that Promotes a Th2-Like Response During *Gallibacterium anatis* Infection in Laying Hens, Veterinary Research. (2020) 51, no. 1, 10.1186/s13567-020-00764-2. PMC7065373 32156313

[27] Kristensen B. M. , Frees D. , and Bojesen A. M. , GtxA from *Gallibacterium anatis*, a Cytolytic RTX-Toxin With a Novel Domain Organisation, Veterinary Research. (2010) 41, no. 3, 10.1051/vetres/2009073, 2-s2.0-84864799139. PMC2820230 19954731

[28] Wang C. , Robles F. , Ramirez S. , Riber A. B. , and Bojesen A. M. , Culture-Independent Identification and Quantification of *Gallibacterium anatis* (*G. anatis*) by Real-Time Quantitative PCR, Avian Pathology. (2016) 45, no. 5, 538–544, 10.1080/03079457.2016.1184743, 2-s2.0-84988423517. 27171757

[29] Sanchez-Alonso P. , Cobos-Justo E. , Avalos-Rangel M. A. et al., A Maverick-Like Cluster in the Genome of a Pathogenic, Moderately Virulent Strain of *Gallibacterium anatis*, ESV200, a Transient Biofilm Producer, Frontiers in Microbiology. (2023) 14, 10.3389/fmicb.2023.1084766. PMC9909271 36778889

